# Recurrence rates and risk factors for seizure recurrence following antiseizure medication withdrawal in adolescent patients with genetic generalized epilepsy

**DOI:** 10.1002/epi4.12603

**Published:** 2022-04-28

**Authors:** Takao Komatsubara, Yu Kobayashi, Akiko Hiraiwa, Shinichi Magara, Moemi Hojo, Takeshi Ono, Kenichi Okazaki, Masafumi Fukuda, Jun Tohyama

**Affiliations:** ^1^ Department of Child Neurology NHO Nishiniigata Chuo Hospital Niigata Japan; ^2^ Department of Pediatrics NHO Niigata Hospital Kashiwazaki Japan; ^3^ Department of Neurosurgery NHO Nishiniigata Chuo Hospital Niigata Japan

**Keywords:** epilepsy with generalized tonic–clonic seizures alone, juvenile absence epilepsy, juvenile myoclonic epilepsy, predictors of recurrence, recurrence rate, valproic acid

## Abstract

**Objective:**

This study aimed to identify the recurrence rate of genetic generalized epilepsy (GGE) and risk factors for recurrence after antiseizure medication (ASM) withdrawal in adolescent patients.

**Methods:**

We retrospectively reviewed medical records of patients with GGE who were included in the registry at the Department of Child Neurology, National Hospital Organization Nishiniigata Chuo Hospital from 2000 through 2020. The eligibility criteria were as follows: onset of epileptic seizures at <15 years of age, treatment with an ASM, and attempted treatment withdrawal at 10‐19 years of age. The rates of seizure recurrence after drug withdrawal were evaluated. Moreover, several variables were evaluated as predictors of recurrence.

**Results:**

In total, 77 patients with GGE (21, 13, and 43 patients with juvenile myoclonic epilepsy [JME], juvenile absence epilepsy [JAE], and epilepsy with generalized tonic–clonic seizures alone [EGTCSA], respectively) were included in this study. Recurrence was detected in 68% of patients with GGE (86%, 31%, and 70% of patients with JME, JAE, and EGTCSA, respectively). Recurrence rates for patients who developed epilepsy at ≥13 years of age, those who started dose reduction at ≥16 years of age, those who exhibited a seizure‐free period of <36 months before withdrawal, and those who chose to discontinue treatment at their own discretion were significantly higher than those for their counterparts. Multivariate analysis revealed that initiation of dose reduction at ≥16 years of age was associated with increased recurrence risk. Meanwhile, a diagnosis of JAE was associated with decreased recurrence risk. All patients with JAE were treated with valproic acid.

**Significance:**

Antiseizure medication withdrawal at ≥16 years of age and a diagnosis other than JAE may be independent risk factors for seizure recurrence after drug withdrawal in adolescent patients.

**Key points**

- The recurrence rate and risk factors for recurrence of GGE after drug withdrawal were retrospectively reviewed for 77 adolescent patients aged 10‐19 years.
- Recurrence was detected in 86%, 31%, and 70% of patients with JME, JAE, and EGTCSA, respectively.
- Antiseizure medication withdrawal at ≥16 years of age and a diagnosis other than JAE were independent risk factors for seizure recurrence after drug discontinuation.
- Antiseizure medication discontinuation should be cautiously approached after thorough discussions with patients and their families.

## 1 INTRODUCTION

Genetic generalized epilepsy (GGE) or idiopathic generalized epilepsy is an epileptic syndrome associated with a genetic predisposition, and it primarily develops in childhood and adolescence.^1^ GGE includes juvenile myoclonic epilepsy (JME), childhood absence epilepsy (CAE), juvenile absence epilepsy (JAE), and epilepsy with generalized tonic–clonic seizures alone (EGTCSA). These diseases are closely related and have overlapping presentations and genetic predispositions.^2^, ^3^, ^4^ CAE is associated with a good prognosis. Administration of appropriate antiseizure medications (ASMs) leads to early seizure remission; ASM dose reduction or discontinuation can also be achieved.^5^, ^6^ Meanwhile, GGE types other than CAE, namely JME and JAE, are associated with a high rate of seizure recurrence after ASM dose reduction or discontinuation following remission.^6^, ^7^ These diseases often require lifelong treatment and affect patients' lives, causing restrictions in schooling, employment, pregnancy, and driver's license acquisition. Thus, when ASM dose reduction or discontinuation is considered achievable, it may be attempted in some patients. Many adolescent patients with GGE request ASM dose reduction or discontinuation; pediatric epileptologists are more likely to consider these requests than adult epileptologists.

Several studies have examined risk factors for recurrence after discontinuation of ASM therapy in patients with childhood epilepsy^8^, ^9^; however, the evidence on risk factors in adolescent patients with GGE remains inconclusive. Recent developments in ASMs have improved the effects of drug therapy; nevertheless, some patients wish to discontinue treatment, and an understanding of recurrence rates and risk factors for recurrence is important to achieve good outcomes in these patients. In addition, this evidence may help counsel adolescent patients with poor drug compliance.

The aim of this study was to evaluate the rate of and risk factors for epileptic seizure recurrence after ASM dose reduction or discontinuation in adolescent patients with GGE.

## 2 PATIENTS AND METHODS

### 2.1 Patients

This retrospective study included patients with GGE (JME, JAE, or EGTCSA) who experienced their first epileptic seizure before 15 years of age and received treatment at the Department of Child Neurology, Epilepsy Center of National Hospital Organization (NHO) Nishiniigata Chuo Hospital from 2000 through 2020, and for whom ASM dose reduction or discontinuation was attempted either at the physician's or patient's discretion (patient age: 10‐19 years) after at least 12 months of remission. Patients with CAE, those who initially developed epileptic syndromes other than GGE, those with ASM withdrawal at ≥20 years of age, and those with ASM withdrawal before a 12‐month remission period were excluded. The diagnosis of an epileptic syndrome was based on the criteria specified in the EpilepsyDiagnosis.org Diagnostic Manual^10^by the International League Against Epilepsy (ILAE).

### 2.2 Methods

The medical records of the patients were retrospectively reviewed. Variables of interest included the following: sex, age at initial onset, age at treatment initiation, age at initiation of dose reduction, type of epilepsy syndrome, duration of the seizure‐free period between the last seizure and the initiation of dose reduction, age at drug discontinuation, duration of the seizure‐free period between the last seizure and drug discontinuation, age at recurrence, timing of recurrence (after or during drug discontinuation), time from drug discontinuation to recurrence, presence or absence of abnormal electroencephalography (EEG) findings before dose reduction, number and types of ASMs used, reasons for ASM dose reduction or discontinuation, and follow‐up duration for patients with or without recurrence after drug withdrawal. The rates of seizure recurrence after drug withdrawal were evaluated. Age (months) was reported as the mean ± standard deviation (SD). We defined generalized epileptiform discharges (either spike–wave discharges or polyspike‐wave discharges) as abnormal EEG findings. Based on the duration of the seizure‐free period between the last seizure and the initiation of dose reduction, the patients were divided into two groups for analysis: <36 months and ≥36 months. The reason for ASM dose reduction or discontinuation was classified as physician‐ (involving discussions with the patient) and patient‐initiated.

The age at epilepsy onset and the initiation of dose reduction, duration of the seizure‐free period until the initiation of dose reduction, presence or absence of abnormal EEG findings, number of drugs used, type of epilepsy syndrome, and reason for ASM dose reduction or discontinuation were evaluated as predictors of recurrence. Selected cut‐off variables for the age at seizure onset, age at the initiation of dose reduction, and seizure‐free period were determined according to the results of previous studies.^11^, ^12^, ^13^ In multivariate analysis, the age at epilepsy onset, age at the initiation of dose reduction, duration of the seizure‐free periods until the initiation of dose reduction, and type of epilepsy syndrome (JAE or JME) were included as independent variables. The reason for ASM dose reduction or discontinuation was excluded from multivariate analysis because it was strongly correlated with the duration of the seizure‐free period.

### 2.3 Statistical analysis

The χ^2^ test and Fisher's exact test were used to compare the variables. A *P*‐value of <.05 was considered statistically significant. The Kaplan‐Meier method and log‐rank test were used to compare seizure recurrence rates among groups defined by the age at seizure onset, age at the initiation of ASM withdrawal, and duration of the seizure‐free periods. Multivariate analysis with the Cox proportional hazard model was used to evaluate the association between different independent variables and the seizure recurrence rate. Univariate analysis was performed to identify independent variables significantly associated with the outcome of interest; these variables were subsequently included in multivariate analysis. All statistical analyses were performed using IBM SPSS Statistics version 24 (IBM Corp.).

### 2.4 Ethics statement

The study protocol adhered to the Declaration of Helsinki and was approved by the ethics committee of the NHO Nishiniigata Chuo Hospital (approval number 20‐21).

## 3 RESULTS

### 3.1 Patient characteristics

**FIGURE 1:**
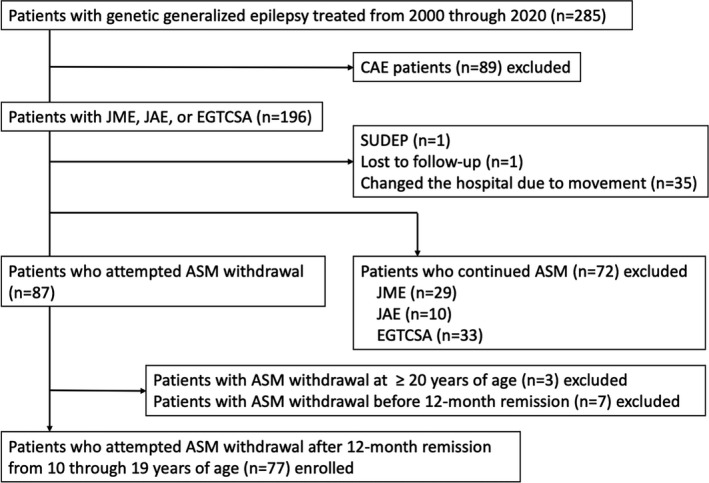
Patient flowchart. In total, 285 patients with GGE syndrome (CAE, JAE, JME, or EGTCSA) were treated at the Department of Child Neurology, Nishiniigata Chuo hospital, from 2000 through 2020. This study excluded patients who initially developed epileptic syndromes other than GGE, patients with CAE, and patients lost to follow‐up. ASM dose reduction or discontinuation was attempted in 87 patients. We excluded patients with ASM withdrawal at ≥20 years of age or a seizure‐free period of <12 months. Finally, 77 patients were included in this study. Abbreviations: ASM, antiseizure medication; CAE, childhood absence epilepsy; EGTCSA, epilepsy with generalized tonic–clonic seizures alone; GGE, genetic generalized epilepsy; JAE, juvenile absence epilepsy; JME, juvenile myoclonic epilepsy; SUDEP, sudden unexpected death in epilepsy

Table 1 presents the demographic and clinical characteristics of the patients. In total, 77 patients with GGE (21, 13, and 43 patients with JME, JAE, and EGTCSA, respectively) were included in this study (Figure 1, Table 1). The average age at seizure onset in patients with JME, JAE, and EGTCSA was 141.5 ± 30.0, 124.8 ± 20.6, and 132.9 ± 30.1 months, respectively. Table 2 presents the ASM types being used at the initiation of dose reduction. Valproic acid was used in 73 of 77 (95%) patients; among them, 61 (79%) patients received valproic acid monotherapy. All patients with JAE received valproic acid.

**TABLE 1:** Demographic and clinical characteristics of all patients with genetic generalized epilepsy and patients with each type of epilepsy syndrome, including those with or without recurrence

GGE syndrome	Sex (M: F)	Age at seizure onset (m ± SD)	Age at ASM treatment (m ± SD)	Age at ASM withdrawal (m ± SD)	Seizure‐free period until ASM withdrawal (m ± SD)	Age at discontinuation of ASM (m ± SD)	Seizure‐free period until discontinuation of ASM (m ± SD)	Recurrence	Age at seizure onset (m ± SD)	Age at recurrence (m ± SD)	Timing of recurrence	Duration from discontinuation till recurrence (m ± SD)	Duration of follow‐up after ASM withdrawal (m ± SD)	Abnormal EEG before tapering (+: −: Unknown)	The number of ASM (one: ≥2)	The reason of ASM withdrawal
JME (n = 21)	8: 13	141.5 ± 30.0	143.6 ± 29.4	192.2 ± 18.0	39.5 ± 14.9	202.6 ± 25.6	54.3 ± 23.2	+ (n = 18)	139.6 ± 33.0	207.4 ± 21.4	After discontinuation (n = 11)	11.5 ± 19.2	109.9 ± 54.9	7: 11: 0	14: 4	3 y remission under doctor‐initiated: 10 patient's initiative: 7 unknown: 1
											During tapering (n = 7)	N.A.				
								− (n = 3)	153.0 ± 2.0	N.A.	N.A.	N.A.	41.3 ± 31.8	0: 3: 0	2: 1	3 y remission under doctor‐initiated: 3
JAE (n = 13)	5: 8	124.8 ± 20.6	132.7 ± 19.9	182.7 ± 24.1	44.8 ± 13.9	195.5 ± 22.5	58.1 ± 14.2	+ (n = 4)	128.6 ± 35.7	215.5 ± 8.3	After discontinuation (n = 3)	5.7 ± 3.9	91.0 ± 65.4	1: 3: 0	2: 2	3 y remission under doctor‐initiated: 3 patient's initiative: 1
											During tapering (n = 1)	N.A.				
								− (n = 9)	123.1 ± 14.1	N.A.	N.A.	N.A.	66.2 ± 25.6	0: 9: 0	7: 2	3 y remission under doctor‐initiated: 9
EGTCSA (n = 43)	28: 15	132.9 ± 30.1	139.8 ± 34.4	189.6 ± 27.5	38.7 ± 14.1	195.1 ± 25.7	47.1 ± 17.3	+ (n = 30)	141.1 ± 27.6	212.4 ± 24.5	After discontinuation (n = 23)	8.4 ± 13.2	106.3 ± 66.3	6: 22: 2	24: 6	3 y remission under doctor‐initiated: 12 patient's initiative: 17 ASM switching: 1
											During tapering (n = 7)	N.A.				
								− (n = 13)	113.2 ± 28.5	N.A.	N.A.	N.A.	41.9 ± 19.2	4: 8: 1	13: 0	3 y remission under doctor‐initiated: 11 patient's initiative: 2
Total (n = 77)	41: 36	133.9 ± 29.2	139.6 ± 31.1	189.1 ± 25.0	39.8 ± 14.6	196.9 ± 25.3	50.9 ± 18.8	+ (n = 52)	139.8 ± 29.7	210.9 ± 22.9	After discontinuation (n = 37)	9.1 ± 14.9 (within 6 mo from discontinuation: 25/37 cases)	106.4 ± 62.7	14: 36: 2	40: 12	3 y remission under doctor‐initiated: 25 patient's initiative: 25 ASM switching: 1 unknown: 1
											During tapering (n = 15)	N.A.				
								− (n = 25)	121.5 ± 25.2	N.A.	N.A.	N.A.	51.3 ± 26.0	4: 20: 1	22: 3	3 y remission with consultation: 23 patient's initiative: 2

Abbreviations: ASM, antiseizure medication; EEG, electroencephalogram; EGTCSA, epilepsy with generalized tonic–clonic seizures alone; F, female; GGE, genetic generalized epilepsy; JAE, juvenile absence epilepsy; JME, juvenile myoclonic epilepsy; M, male; mo, month; N.A., not applicable; SD, standard deviation; y—year.

**TABLE 2:** Antiseizure medication at the time of withdrawal

	ASMs	No.
JME (n = 21)	VPA	16
	VPA + LTG	2
	VPA + ESM	1
	CZP + CLB	1
	PB + ZNS	1
JAE (n = 13)	VPA	9
	VPA + AZA	1
	VPA + CZP	1
	VPA + ESM	1
	VPA + LTG	1
EGTCSA (n = 43)	VPA	36
	VPA + ZNS	2
	VPA + CBZ	1
	VPA + CLB	1
	VPA + CZP	1
	CBZ	1
	CZP + AZA	1

Abbreviations: ASM, antiseizure medication; AZA, acetazolamide; CBZ, carbamazepine; CLB, clobazam; CZP, clonazepam; EGTCSA, epilepsy with generalized tonic–clonic seizures alone; ESM, ethosuximide; JAE, juvenile absence epilepsy; JME, juvenile myoclonic epilepsy; LTG, lamotrigine; PB, phenobarbital; VPA, valproic acid; ZNS, zonisamide.

### 3.2 Rate of seizure recurrence

Table 1 presents the number of characteristics of recurrence for each GGE type. Recurrence was observed in 52 of 77 (68%) patients with GGE, including 18 of 21 (86%) patients with JME, 4 of 13 (31%) patients with JAE, and 30 of 43 (70%) patients with EGTCSA (Table 1). The mean age at onset of seizures for patients with and those without recurrence in the JME, JAE, and EGTCSA groups was 139.6 ± 33.0 and 153.0 ± 2.0 months, 128 ± 35.7 and 123.1 ± 14.1 months, and 141.1 ± 27.6 and 113.2 ± 28.5 months, respectively. The mean follow‐up duration after ASM withdrawal for patients with and without recurrence was 106.4 ± 62.7 and 51.3 ± 26.0 months, respectively (*P* < .001).

### 3.3 Risk factors for recurrence

**FIGURE 2:**
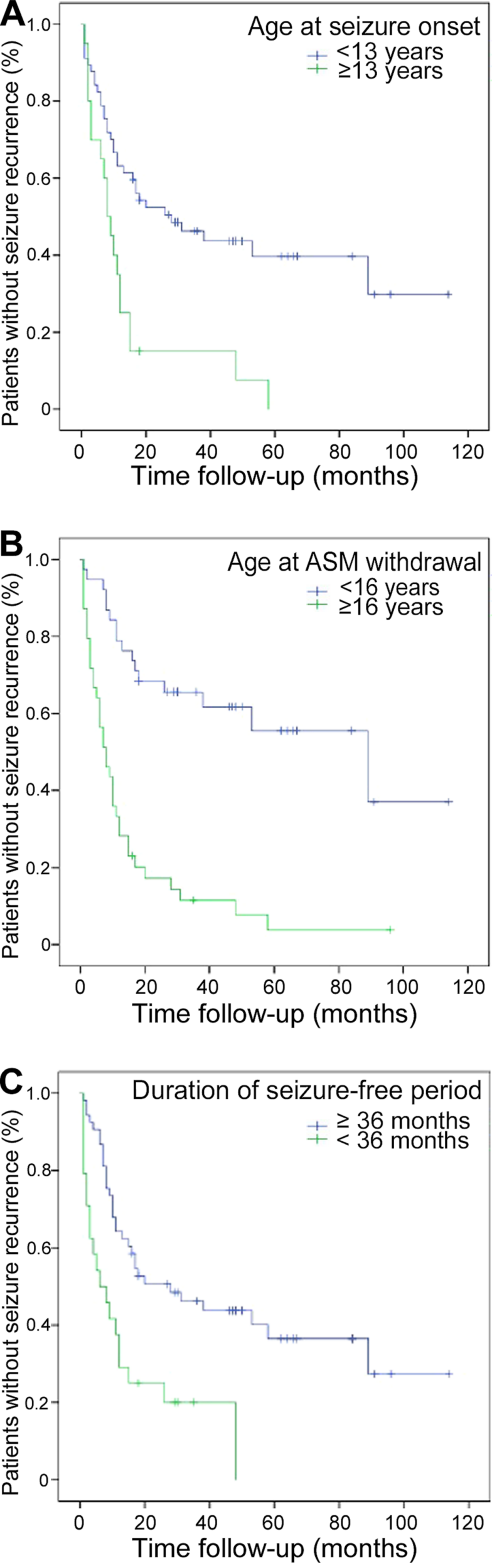
Cumulative probability of seizure recurrence rate for each risk factor. (A) Age at seizure onset. (B) Age at ASM withdrawal. (C) Duration of the seizure‐free period until ASM withdrawal. Abbreviation: ASM, antiseizure medication

Table 3 presents a comparison of the recurrence rates among predictors. Seizure onset at ≥13 years of age (odds ratio [OR], 13.82; 95% confidence interval [CI], 1.729‐110.440; *P* = .002), initiation of ASM dose reduction at ≥16 years of age (OR, 16.50; 95% CI, 4.31‐63.162; *P* < .001), a seizure‐free period of <36 months until ASM withdrawal (OR, 3.28; 95% CI, 0.982‐10.964; *P* = .046), and patient‐initiated withdrawal (OR, 11.5; 95% CI, 2.447‐54.052; *P* < .001) were associated with an increased risk of seizure recurrence. The Kaplan‐Meier curves for seizure recurrence rates associated with the age at seizure onset (Figure 2A), age at the initiation of ASM dose reduction (Figure 2B), and seizure‐free period before ASM withdrawal (Figure 2C) are presented. Abnormal EEG findings, the number of ASMs used, and sex were not associated with the risk of seizure recurrence. The recurrence rate for JME was higher than the combined recurrence rate for the other two types (*P* = .037), whereas the recurrence rate for JAE was lower than the combined recurrence rate for the other two types (*P* = .02). The recurrence rate for EGTCSA did not differ from the combined recurrence rate for the other two types.

**TABLE 3:** Clinical risk factors for seizure recurrence

Predictor		The χ^2^ test or Fisher's exact test		Multivariate analysis	
	Recurrence rate cases (%)	Odds ratio (95% CI)	*P*‐value	Hazard ratio (95% CI)	*P*‐value
Age at seizure onset (mean seizure‐free period until ASM withdrawal)					
13 y and over (30.6 mo)	19/20 (95)	13.82 (1.729‐110.440)	.002	0.880 (0.432‐1.792)	.725
13 y > (43.1 mo)	33/57 (58)				
Age at ASM withdrawal (mean seizure‐free period until ASM withdrawal)					
16 y and over (38.9 mo)	36/39 (92)	16.5 (4.310‐63.162)	<.001	4.097 (2.052‐8.182)	<.001
16 y > (40.8 mo)	16/38 (42)				
Seizure‐free period until ASM withdrawal					
36 mo and over	32/53 (60)	3.281 (0.982‐10.964)	.046	1.591 (0.821‐3.083)	.169
36 mo>	20/24 (83)				
Abnormal EEG before tapering					
+	14/18 (78)	1.944 (0.564‐6.708)	.287	—	—
−	36/56 (64)				
The number of ASM					
2 and more	12/15 (80)	2.20 (0.560‐8.640)	.202	—	—
1	40/62 (65)				
Sex					
M	29/41 (71)	1.366 (0.525‐3.555)	.522	—	—
F	23/36 (64)				
Epilepsy syndrome					
JME	18/21 (86)	3.882 (1.022‐14.749)	.037	—	—
JAE	4/13 (31)	0.148 (0.040‐0.547)	.003	—	—
EGTCSA	30/43 (70)	1.259 (0.483‐3.282)	.638	—	—
Other than JME		—	—	1.195 (0.648‐2.201)	.586
Other than JAE		—	—	3.389 (1.126‐10.201)	.03
The reason of ASM withdrawal					
3 y seizure‐free period with consultation	25/48 (52)	11.500 (2.447‐54.052)	<.001	—	—
Patient's initiative	25/27 (93)				

Abbreviations: ASM, antiseizure medication; CI, confidence interval; EEG, electroencephalogram; EGTCSA, epilepsy with generalized tonic–clonic seizures alone; F, female; JAE, juvenile absence epilepsy; JME, juvenile myoclonic epilepsy; M, male; mo, month; n.s., not significant; y, year.

Multivariate Cox proportional hazard regression analysis revealed that initiation of dose reduction at ≥16 years of age and a diagnosis other than JAE were independent risk factors for recurrence (hazard ratio [HR], 4.095; 95% CI, 2.052‐8.083; *P* < .001 and HR, 3.389; 95% CI, 1.126‐10.201; *P* < .03, respectively; Table 3).

## 4 DISCUSSION

### 4.1 Seizure recurrence rate

In the present study, seizure recurrence was observed in 52 of 77(68%) patients with GGE. The recurrence rates for patients with JME, JAE, and EGTCSA were 86%, 31%, and 70%, respectively. Table 4 compares the rates in the present study with previously reported rates,^14^, ^15^, ^16^, ^17^, ^18^, ^19^ some of which were relatively low.^14^, ^15^, ^16^ The low recurrence rates in previous studies may be attributed to several factors. First, the period from the last seizure to dose reduction was ≥3 years, which is considerably long. Second, one study only included patients without deterioration of EEG findings during dose reduction,^14^ while another included patients with a mean age of 6.7 years at epilepsy onset.^16^ Third, seizures were controlled by one or two ASMs in most (86%) patients.

**TABLE 4:** Recurrence rates for genetic generalized epilepsy in previous and present studies

First author	Seizure‐free period until ASM withdrawal (mean)	Recurrence rate (cases)				Remarkable notes
		GGE	JME	JAE	EGTCSA	
Yamatani M^14^	3 y	31.4% (11/35)	—	—	—	GGEs include JME, JAE, and EGTCSA. The number of each GGE syndrome was not described. Normal EEG at ASM withdrawal
Vorderwülbecke BJ^15^	ND	51.8% (29/56)	21.4% (3/14)	66.7% (10/15)	59.3% (16/27)	Median duration of remission. (JME: 6 y, JAE: 12.5 y, EGTCSA: 8 y)
Camfield P^16^	3.5 y	—	—	—	18.1% (6/33)	Mean age at seizure onset was 6.7 y Most patients (86%) ware managed one or two ASM
Pavlović M^17^	ND	78.6% (22/28)	100% (10/10)	50% (4/8)	80% (8/10)	Median duration of remission was 4 y Median age at ASM withdrawal was 14.3 y (including 21 CAE patients)
Healy L^18^	ND	—	80% (8/10)	100% (6/6)	—	Duration of remission was 2 y at the least
Irelli EC^19^	ND	100% (3/3)				Adult‐onset GGE
This study	GGE (total):39.8 mo JME:39.5 mo JAE:44.8 mo EGTCSA:38.7 mo	68% (52/77)	86% (18/21)	31% (4/13)	70% (30/43)	—

Abbreviations: ASM, antiseizure medication; CAE, childhood absence epilepsy; EEG, electroencephalogram; EGTCSA, epilepsy with generalized tonic–clonic seizures alone; GGE, genetic generalized epilepsy; JAE, juvenile absence epilepsy; JME, juvenile myoclonic epilepsy; mo, month; ND, not described; y, year.

In contrast, some previous studies reported high recurrence rates,^17^, ^18^, ^19^ which may be attributed to several factors. A study by Pavlović et al^17^ included patients with a median age of 14.3 years at the initiation of dose reduction; that study examined 59 patients with GGE, including 21 with CAE. If patients with CAE, which often occurs at a young age, were excluded, the mean age at the initiation of dose reduction might have been higher. In a study by Healy et al,^18^ the duration of remission until the initiation of dose reduction was relatively short, with a minimum of 2 years. Irelli et al^19^ attempted ASM withdrawal in three patients with adult‐onset GGE, all of whom experienced disease recurrence. The present findings cannot be directly compared with the findings in these previous studies because of differences in seizure‐free periods, criteria for dose reduction, and study exclusion criteria; however, the recurrence rates for JME and EGTCSA in the present study can be considered comparable with those in the previous reports.

The recurrence rate for JAE was lower in the present study than that in previous studies.^18^ In multivariate analysis, a diagnosis of JAE was significantly associated with a low risk of recurrence. This study used the EpilepsyDiagnosis.org Diagnostic Manual by ILAE^10^ for the diagnosis of epilepsy, including JAE. According to this manual, JAE should be differentiated from CAE according to the frequency of epileptic seizures in patients who develop seizures at 8 to 12 years of age. In some cases, this distinction is difficult. Among pediatric patients with epilepsy who develop generalized tonic–clonic seizures, younger patients who meet the diagnostic criteria for EGTCSA are less likely to experience recurrence after drug withdrawal than are older patients.^16^, ^20^ Thus, even among patients with JAE, those with a younger age at onset may exhibit a better prognosis than do their counterparts. The low recurrence rate for JAE in the present study may be attributed to the fact that the included patients were relatively young at disease onset, the period between disease remission and drug discontinuation was relatively long, and only one patient showed abnormal EEG findings before dose reduction. However, the present study revealed no difference in the mean age at onset between patients with JAE with recurrence and those without recurrence.

In the present study, the mean follow‐up period after ASM withdrawal was shorter for patients without recurrence than for those with recurrence, probably because patients without recurrence usually discontinued their follow‐up visits after an appropriate duration. The mean follow‐up period after ASM withdrawal was 51.3 months for patients without recurrence. The mean duration from ASM discontinuation to recurrence was 9.1 months for patients with recurrence, and 15 patients showed seizure relapse during ASM dose reduction. Although the follow‐up period was shorter for patients without recurrence, the mean follow‐up period of ≥4 years was adequate given the timing of recurrence in patients with recurrence.

### 4.2 Risk factors for seizure recurrence

In the present study, the age at epilepsy onset and initiation of dose reduction were risk factors for recurrence (Table 3). Recurrence rates were significantly higher for patients who developed epilepsy at ≥13 years of age than for those who developed epilepsy at <13 years of age (*P* = .002; Figure 2A). In addition, these rates were significantly higher for patients who underwent dose reduction at ≥16 years of age than for those who underwent dose reduction at <16 years of age (*P* < .001; Figure 2B). Multivariate analysis showed that initiation of dose reduction at ≥16 years of age was associated with an increased risk of recurrence. A previous study reported that the relative risk of recurrence was 1.79‐fold and 1.34‐fold higher for patients with adolescent‐ and adult‐onset epilepsy (age of onset ≥20 years), respectively, than for those with childhood‐onset epilepsy.^11^ In addition, the Medical Research Council Antiepileptic Drug Withdrawal Study Group (MRC study)^13^ reported that the risk of recurrence was 1.75‐fold higher when drug dose reduction was initiated at ≥16 years of age than that at a younger age. The present findings are in line with those of previous studies on adolescent patients with epilepsy.

In the present study, the duration of the seizure‐free period until ASM withdrawal was a risk factor for recurrence. Recurrence was detected in 20 of 24 patients with a seizure‐free period of <36 months and in 32 of 53 patients with a seizure‐free period of ≥36 months (Table 3, Figure 2C). The MRC study^21^ reported the rate of seizure recurrence after discontinuation of ASMs for 1013 patients, including adults and children whose seizures were controlled for ≥2 years. Compared with those for patients with a seizure‐free period of <2.5 years between the last seizure and the initiation of dose reduction, the risk ratios for recurrence for patients with seizure‐free periods of 2.5‐3, 3‐5, 5‐10, and ≥10 years were 0.94, 0.67, 0.47, and 0.27, respectively. This suggests that the recurrence rate may decrease with an increase in the seizure‐free period. Strozzi et al^22^ performed a meta‐analysis of studies that included 924 patients with epilepsy who were aged <16 years and showed that the relative risk of recurrence for patients with early drug discontinuation after a seizure‐free period of <2 years versus patients with a seizure‐free period of ≥2 years was 1.34 (95% CI, 1.13‐1.59; *P* = .0007). Recently, Contento et al^23^ reported that a drug‐free period of <2 years was the main predictor of seizure recurrence. In our study, Fisher's exact test revealed that the recurrence rate was higher for patients with a seizure‐free period of <3 years than for their counterparts (*P* = .046). However, in multivariate analysis, the duration of the seizure‐free period was not a significant risk factor for recurrence; in fact, initiation of dose reduction at ≥16 years of age was a risk factor. When patients who develop epilepsy at 13 years of age exhibit a seizure‐free period of 3 years, they inevitably initiate dose reduction at ≥16 years of age. Some reports have suggested that a seizure‐free period of 2 years is acceptable. The treatment of pediatric patients with epilepsy requires thorough disease status assessments, and it should be carefully confirmed if ASM doses can be reduced in patients below 16 years of age.

In addition, in the present study, the seizure recurrence rate was lower for patients who discontinued ASM in consultation with a physician after a seizure‐free period of 3 years than for those who reduced their doses at their own discretion. Overall, evidence suggests that a seizure‐free period of ≥3 years may be an appropriate criterion for drug discontinuation in adolescent patients with GGE. However, completion of drug therapy may be difficult for patients with dose reduction at ≥16 years of age. Thus, patients and their families should be thoroughly counseled with regard to drug compliance, lifestyle choices, and other factors that may affect outcomes. Given the high recurrence rates for patients with JME and EGTCSA, decisions about treatment discontinuation should include through clinical assessments and discussions with patients and their families.

The present study detected recurrence in 14 of 18 (78%) patients with abnormal EEG findings before ASM dose reduction. All patients with JME (n = 7) showing abnormal EEG findings experienced recurrence, whereas recurrence was detected in 36 of 56 (64%) patients without abnormal EEG findings. No significant difference in outcomes was observed. These results may be attributed to the insufficient sample size for the detection of significant predictors of recurrence. It is plausible that EEG abnormalities do not affect the risk of recurrence. Moreover, recurrence rates tended to be relatively high for patients treated with multiple ASMs. However, the number of prescribed ASM did not affect the outcome. Further studies should include larger samples to identify risk factors for epilepsy recurrence in patients who terminate their treatment.

### 4.3 Timing of seizure recurrence

In the present study, 40 of 52 (77%) patients with recurrence developed recurrence during dose reduction or within 6 months after drug discontinuation (Table 1). Pavlović et al^17^ evaluated recurrence rates after drug discontinuation in 44 patients with GGE, including 16 with CAE. They observed that 20% of patients exhibited recurrence during dose reduction, while 54.5% exhibited it within 6 months after drug discontinuation. The authors concluded that recurrence is likely to occur during dose reduction and within 6 months after drug discontinuation in 50% patients. The present findings were similar, suggesting that patients should be monitored closely in the period immediately after drug discontinuation, when the risk of recurrence is the highest.

### 4.4 Treatment of GGE and recurrence after ASMs withdrawal in adolescent patients

In the present study, many patients were treated with valproic acid rather than with modern ASM. Consequently, we did not evaluate recurrence rates according to the ASM types. This may be attributed to the delayed introduction of new ASMs in Japan or the inclusion of registered cases treated before these compounds were available. Valproic acid is not recommended for women of reproductive age,^24^ and more modern drugs have been used for both initial treatment and as alternatives to valproic acid. On the one hand, seizure control rates associated with valproic acid and levetiracetam are reportedly similar for patients with generalized epilepsy.^25^, ^26^ On the other hand, some studies have shown that the seizure control rate is higher with valproic acid than with new ASMs in patients with JME.^27^, ^28^ The second Standard and New Antiepileptic Drugs study, which compared valproic acid and levetiracetam, has also demonstrated the superiority of valproic acid.^29^ Furthermore, a study has shown that ethosuximide and valproic acid are more effective than lamotrigine in the treatment of absence seizures,^30^ and it recommended the use of ethosuximide or valproic acid for initial treatment. For patients with concomitant generalized tonic–clonic seizures, valproic acid has been reported to be more effective than ethosuximide.^31^ The diagnosis of JAE is associated with low recurrence risk. Many patients in this study were treated with valproic acid, which may have effectively reduced seizure recurrence in young patients with GGE, specifically JAE. Further studies are required to confirm these findings.

### 4.5 Limitations

The present study had several limitations. First, it was a retrospective study; consequently, there was no dose control and patients received different ASM doses. Second, the sample size may have been insufficient to detect significant effects on the risk factors on the recurrence rates. Third, the included patients did not form a population‐based cohort but were selected from the registry of the pediatric department at a single regional epilepsy center. Consequently, our patient cohort did not reflect the real‐world distribution of patients with GGE in this region. Fourth, most patients treated at this epilepsy center are referred from other hospitals for surgical treatment; thus, the present study may have included difficult‐to‐treat cases. Fifth, the included patients were relatively young; the age distribution in this study sample cannot be generalized to all patients with GGE. Finally, ASM withdrawal was attempted only for patients who wished to attempt it at their own discretion or in consultation with a physician. Consequently, the distribution of patients who attempted dose reduction was not balanced, and the duration of the seizure‐free period was inconsistent. Thus, the present findings cannot be generalized to all patients with GGE who discontinue treatment at any time.

## 5 CONCLUSION

In the present study, the recurrence rates for patients with JME and EGTCSA were comparable with previously reported rates, whereas those for patients with JAE were lower than previously reported rates. Initiation of ASM dose reduction at ≥16 years of age was associated with an increased risk of recurrence, whereas a diagnosis of JAE was associated with a low risk of recurrence. Completion of drug therapy may be difficult for patients with dose reduction at ≥16 years of age. The decision to reduce ASM doses in patients with childhood‐onset GGE, particularly those <16 years of age should be based on the disease and treatment status and be done in consultation with patients and their families.

## CONFLICT OF INTEREST

Jun Tohyama has received speaker honoraria from UCB Japan, Daiichi‐Sankyo, Eisai, BioMarin, Novartis pharma, and LivaNova and is an investigator in clinical trials sponsored by UCB Japan, Zogenix, and Takeda (no personal compensation). Yu Kobayashi has received speaker honoraria from Novartis pharma and BioMarin. Shinichi Magara has received speaker honoraria from Chugai Pharmaceutical. Masafumi Fukuda has received speaker honoraria from UCB Japan, Daiichi‐Sankyo, Eisai, Novartis pharma, and LivaNova and is an investigator in clinical trials sponsored by UCB Japan (no personal compensation). The remaining authors have no conflicts of interest. We confirm that we have read the journal's position on issues involved in ethical publication and affirm that this report is consistent with those guidelines.

## References

[1] Scheffer IE , Berkovic S , Capovilla G , Connolly MB , French J , Guilhoto L , et al. ILAE classification of the epilepsies: position paper of the ILAE Commission for Classification and Terminology. Epilepsia. 2017;58:512–21. 28276062 10.1111/epi.13709 PMC5386840

[2] Janz D . The idiopathic generalized epilepsies of adolescence with childhood and juvenile age of onset. Epilepsia. 1997;38:4–11. 9024180 10.1111/j.1528-1157.1997.tb01073.x

[3] Marini C , Scheffer IE , Crossland KM , Grinton BE , Phillips FL , McMahon JM , et al. Genetic architecture of idiopathic generalized epilepsy: clinical genetic analysis of 55 multiplex families. Epilepsia. 2004;45:467–78. 15101828 10.1111/j.0013-9580.2004.46803.x

[4] Greenberg DA , Durner M , Resor S , Rosenbaum D , Shinnar S . The genetics of idiopathic generalized epilepsies of adolescent onset: differences between juvenile myoclonic epilepsy and epilepsy with random grand mal and with awakening grand mal. Neurology. 1995;45:942–6. 7746411 10.1212/wnl.45.5.942

[5] Grosso S , Galimberti D , Vezzosi P , Farnetani M , Di Bartolo RM , Bazzotti S , et al. Childhood absence epilepsy: evolution and prognostic factors. Epilepsia. 2005;46:1796–801. 16302860 10.1111/j.1528-1167.2005.00277.x

[6] Camfield C , Camfield P . Management guidelines for children with idiopathic generalized epilepsy. Epilepsia. 2005;46(Suppl 9):112–6. 16302884 10.1111/j.1528-1167.2005.00322.x

[7] Seneviratne U , Cook M , D'Souza W . The prognosis of idiopathic generalized epilepsy. Epilepsia. 2012;53:2079–90. 23106474 10.1111/j.1528-1167.2012.03723.x

[8] Verrotti A , D'Egidio C , Agostinelli S , Parisi P , Spalice A , Chiarelli F , et al. Antiepileptic drugs withdrawal in childhood epilepsy: what are the risk factors associated with seizure relapse? Eur J Pediatr Neurol. 2012;16:599–604. 10.1016/j.ejpn.2012.02.002 22398177

[9] Karalok ZS , Guven A , Öztürk Z , Gurkas E . Risk factors for recurrence after drug withdrawal in childhood epilepsy. Brain and Development. 2020;42:35–40. 31521420 10.1016/j.braindev.2019.08.012

[10] EpilepsyDiagnosis.org . Diagnostic manual. https://www.epilepsydiagnosis.org. Accessed January 23, 2022.

[11] Berg AT , Shinnar S . Relapse following discontinuation of antiepileptic drugs: a meta‐analysis. Neurology. 1994;44:601–8. 8164811 10.1212/wnl.44.4.601

[12] Shinnar S , Berg AT , Moshé SL , Kang H , O'Dell C , Alemany M , et al. Discontinuing antiepileptic drugs in children with epilepsy: a prospective study. Ann Neurol. 1994;35:534–45. 8179299 10.1002/ana.410350506

[13] Medical Research Council Antiepileptic Drug Withdrawal Study Group . Prognostic index for recurrence of seizures after remission of epilepsy. BMJ. 1993;306:1374–8. 8518603 10.1136/bmj.306.6889.1374 PMC1677783

[14] Yamatani M , Konishi T , Matsuzawa J , Hongou K , Yagi S . Relapse of seizure after withdrawal of antiepileptic drug treatment in childhood epilepsy: age‐dependent factors. No To Hattatsu. 2000;32:15–20. 10655745

[15] Vorderwülbecke BJ , Kowski AB , Kirschbaum A , Merkle H , Senf P , Janz D , et al. Long‐term outcome in adolescent‐onset generalized genetic epilepsies. Epilepsia. 2017;58:1244–50. 28464258 10.1111/epi.13761

[16] Camfield P , Camfield C . Idiopathic generalized epilepsy with generalized tonic‐clonic seizures (IGE‐GTC): a population‐based cohort with >20 year follow up for medical and social outcome. Epilepsy Behav. 2010;18:61–3. 20471324 10.1016/j.yebeh.2010.02.014

[17] Pavlović M , Jović N , Pekmezović T . Antiepileptic drugs withdrawal in patients with idiopathic generalized epilepsy. Seizure. 2011;20:520–5. 21493107 10.1016/j.seizure.2011.03.007

[18] Healy L , Moran M , Singhal S , O'Donoghue MF , Alzoubidi R , Whitehouse WP . Relapse after treatment withdrawal of antiepileptic drugs for juvenile absence epilepsy and juvenile myoclonic epilepsy. Seizure. 2018;59:116–22. 29807291 10.1016/j.seizure.2018.05.015

[19] Irelli EC , Orlando B , Salamone EM , Fisco G , Barone FA , Morano A , et al. High rates of early remission pattern in adult‐onset compared with earlier‐onset idiopathic generalized epilepsy: a long‐term follow‐up study. Seizure. 2022;94:52–6. 34864252 10.1016/j.seizure.2021.11.019

[20] Caraballo R , Silva S , Beltran L , Calvo A , Caballero R . Childhood‐only epilepsy with generalized tonic‐clonic seizures: a well‐defined epileptic syndrome. Epilepsy Res. 2019;153:28–33. 30947078 10.1016/j.eplepsyres.2019.03.017

[21] Medical Research Council Antiepileptic Drug Withdrawal Study Group . Randomised study of antiepileptic drug withdrawal in patients in remission. Lancet. 1991;337(8751):1175–80. 1673736

[22] Strozzi I , Nolan SJ , Sperling MR , Wingerchuk DM , Sirven J . Early versus late antiepileptic drug withdrawal for people with epilepsy in remission. Cochrane Database Syst Rev. 2015;2015(2):CD001902. 10.1002/14651858.CD001902.pub2 PMC7061653 25922863

[23] Contento M , Bertaccini B , Biggi M , Magliani M , Failli Y , Rosati E , et al. Prediction of seizure recurrence risk following discontinuation of antiepileptic drugs. Epilepsia. 2021;62:2159–70. 34250596 10.1111/epi.16993 PMC8457060

[24] European Medicines Agency . New measures to avoid valproate exposure in pregnancy endorsed. EMA/145600/2018; 2018. https://www.ema.europa.eu/en/documents/press‐release/new‐measures‐avoid‐valproate‐exposure‐pregnancy‐endorsed_en.pdf. Accessed January 23, 2022.

[25] Tabrizi N , Zarvani A , Rezaei P , Cheraghmakani H , Alizadeh‐Navaei R . Levetiracetam in genetic generalized epilepsy: a prospective unblinded active‐controlled trial. Epilepsy Res. 2019;157:106214. 31627041 10.1016/j.eplepsyres.2019.106214

[26] Abdelmesih SK , Elkhateeb N , Zakaria M , Girgis MY . Initial levetiracetam versus valproate monotherapy in antiseizure medicine (ASM)‐naïve pediatric patients with idiopathic generalized epilepsy with tonic‐clonic seizures. Seizure. 2021;91:263–70. 34246881 10.1016/j.seizure.2021.06.033

[27] Silvennoinen K , de Lange N , Zagaglia S , Balestrini S , Androsova G , Wassenaar M , et al. Comparative effectiveness of antiepileptic drugs in juvenile myoclonic epilepsy. Epilepsia Open. 2019;4:420–30. 31440723 10.1002/epi4.12349 PMC6698679

[28] Irelli EC , Morano A , Cocchi E , Casciato S , Fanella M , Albini M , et al. Doing without valproate in women of childbearing potential with idiopathic generalized epilepsy: implications on seizure outcome. Epilepsia. 2020;61:107–14. 31828782 10.1111/epi.16407

[29] Marson A , Burnside G , Appleton R , Smith D , Leach JP , Sills G , et al. The SANAD II study of the effectiveness and cost‐effectiveness of valproate versus levetiracetam for newly diagnosed generalised and unclassifiable epilepsy: an open‐label, non‐inferiority, multicentre, phase 4, randomised controlled trial. Lancet. 2021;397:1375–86. 33838758 10.1016/S0140-6736(21)00246-4 PMC8047813

[30] Glauser TA , Cnaan A , Shinnar S , Hirtz DG , Dlugos D , Masur D , et al. Ethosuximide, valproic acid, and lamotrigine in childhood absence epilepsy: initial monotherapy outcomes at 12 months. Epilepsia. 2013;54:141–55. 23167925 10.1111/epi.12028 PMC3538883

[31] Brigo F , Igwe SC , Lattanzi S . Ethosuximide, sodium valproate or lamotrigine for absence seizures in children and adolescents. Cochrane Database Syst Rev. 2019;2:CD003032. 30734919 10.1002/14651858.CD003032.pub4 PMC6367681

